# Aging Brain and Hearing: A Mini-Review

**DOI:** 10.3389/fnagi.2021.791604

**Published:** 2022-01-13

**Authors:** Yasue Uchida, Yukiko Nishita, Rei Otsuka, Saiko Sugiura, Michihiko Sone, Tatsuya Yamasoba, Takashi Kato, Kaori Iwata, Akinori Nakamura

**Affiliations:** ^1^Department of Otolaryngology, Aichi Medical University, Nagakute, Japan; ^2^Department of Otorhinolaryngology, National Center for Geriatrics and Gerontology, Obu, Japan; ^3^Department of Epidemiology of Aging, Center for Gerontology and Social Science, National Center for Geriatrics and Gerontology, Obu, Japan; ^4^Section of NILS-LSA, Center for Gerontology and Social Science, National Center for Geriatrics and Gerontology, Obu, Japan; ^5^Toyota Josui Mental Clinic, Toyota, Japan; ^6^Department of Otorhinolaryngology, Nagoya University Graduate School of Medicine, Nagoya, Japan; ^7^Department of Otolaryngology-Head and Neck Surgery, Faculty of Medicine, Graduate School of Medicine, The University of Tokyo, Tokyo, Japan; ^8^Department of Clinical and Experimental Neuroimaging, National Center for Geriatrics and Gerontology, Obu, Japan

**Keywords:** hearing, brain reserve, brain volume, magnetic resonance imaging, hippocampus

## Abstract

Brain reserve is a topic of great interest to researchers in aging medicine field. Some individuals retain well-preserved cognitive function until they fulfill their lives despite significant brain pathology. One concept that explains this paradox is the reserve hypothesis, including brain reserve that assumes a virtual ability to mitigate the effects of neuropathological changes and reduce the effects on clinical symptoms flexibly and efficiently by making complete use of the cognitive and compensatory processes. One of the surrogate measures of reserve capacity is brain volume. Evidence that dementia and hearing loss are interrelated has been steadily accumulating, and age-related hearing loss is one of the most promising modifiable risk factors of dementia. Research focused on the imaging analysis of the aged brain relative to auditory function has been gradually increasing. Several morphological studies have been conducted to understand the relationship between hearing loss and brain volume. In this mini review, we provide a brief overview of the concept of brain reserve, followed by a small review of studies addressing brain morphology and hearing loss/hearing compensation, including the findings obtained from our previous study that hearing loss after middle age could affect hippocampal and primary auditory cortex atrophy.

## Introduction

With the increase in aging population globally, dementia is a rapidly growing public health problem. While there is no fundamentally curative treatment, the proactive management of modifiable risk factors or resilience, which can delay the progression or slow down the onset of the disease, has become a focus of research in aging medicine field.

**FIGURE 1:**
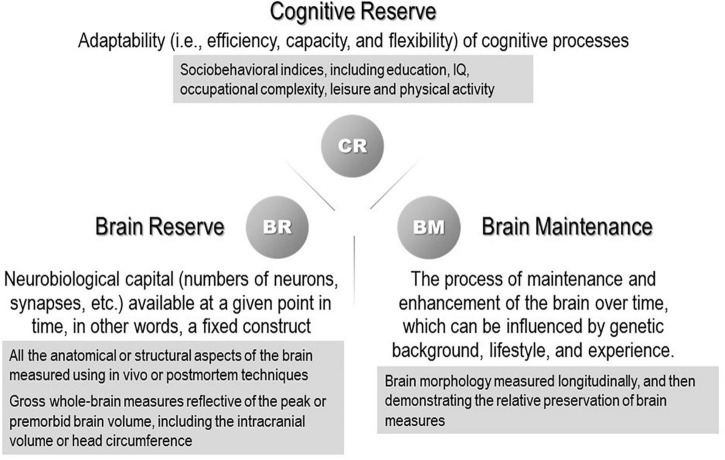
Concepts and definitions of reserve-related terms (Stern et al., 2020). (The proxy measures used in previous studies are shown in the gray boxes.)

The susceptibility to developing dementia varies greatly across individuals, and individual differences are still not well understood. Several concepts that account for “resilience” to aging and associated pathological changes have been the subject of numerous studies. Some individuals retain well-preserved cognitive function until they fulfill their lives despite significant brain pathology. One concept that explains this paradox is the reserve hypothesis that assumes a virtual ability to mitigate the effects of neuropathological changes and reduce the effects on clinical symptoms flexibly and efficiently by making complete use of the cognitive and compensatory processes. “Resilience” is a general term referring to multiple reserve-related processes, including the concepts of brain reserve, cognitive reserve, and brain maintenance. Their definitions were recently organized in a whitepaper published by the Reserve, Resilience and Protective Factors PIA Empirical Definitions and Conceptual Framework Workgroup under the auspices of the Alzheimer’s Association to develop a consensus (Stern et al., 2020; Figure 1). In the whitepaper, brain reserve was colloquially likened to the “hardware,” whereas cognitive reserve was the “software.” The multiple constructs of resilience that scientists have invoked are all theoretical entities and currently cannot be measured directly. The surrogate measures of brain reserve capacity are all anatomical or structural aspects of the brain.

Since the 2017 report of the Lancet International Commission on Dementia Prevention, Intervention, and Care (Livingston et al., 2017), in which hearing loss was estimated to be the largest contributor to potentially modifiable risk factors for dementia, it has received high attention in the field of aging. Hearing loss still ranks at the top of this list in the latest report released in 2020 (Livingston et al., 2020). The impact of hearing loss as a dementia risk was reported socially with surprise since it was not a risk factor that had long been identified. The Lancet International Commission has used the estimate model with the population attributable fraction, which is the proportional (percentage) reduction in new cases of dementia that would occur if specific risk factors were eliminated; therefore, the high prevalence of hearing loss should be reflected.

In this mini review, we provide a brief overview of the concept of brain reserve, followed by a small review of studies addressing brain morphology and hearing loss/hearing compensation.

## Brain Reserve

The concept of brain reserve has been introduced to explain the fact that patients with Alzheimer’s disease (AD) who met the neuropathological criteria for AD with high brain volume generally have good clinical outcomes (Katzman et al., 1988). Katzman et al. (1988), performed a study of 137 residents (average age 85.5 years) of a nursing facility whose mental status, memory, and functional status had been evaluated during life, and described a group of individuals in whom there was a discrepancy between the functional assessment while alive and AD pathology assessment obtained by postmortem examination. The individuals showed a marked presence of neuritic plaques and neurofibrillary tangles but were classified as clinically non-demented and their brains were characterized by a high weight and number of neurons. Katzman et al. (1988), hypothesized that a large brain size may be protective against the clinical expression of pathology and that it might have high reserve.

Stern discerned brain reserve from cognitive reserve and refined the definition of brain reserve (Stern, 2012; Stern et al., 2019). Brain reserve is commonly estimated using the intracranial volume (ICV), which is an easily accessible measure obtained from structural magnetic resonance imaging (MRI) (van Loenhoud et al., 2018; Stern et al., 2020). Brain reserve should be measured in a quantitative manner, including the number of neurons or synapses and/or dendritic spines (Boros et al., 2017). van Loenhoud et al. (2018), evaluated the validity of using the ICV as a proxy for brain reserve in a meta-analysis of 10 studies and showed that a higher ICV was associated with higher cognitive performance after adjusting for the presence and amount of pathology. While acknowledging that ICV is not necessarily a straightforward measure of total brain capacity, van Loenhoud et al. (2018), concluded that the use of ICV as a proxy for brain reserve was justified at the time because it captured several aspects of brain reserve.

Brain size is largely determined by biological and genetic backgrounds (Bartley et al., 1997; Jansen et al., 2020). Although the extent to which genetic and environmental factors contribute to individual differences is unclear, the relative contributions of these factors to the brain structure vary over lifetime (Batouli et al., 2014). Even after adulthood, unlike most regions of the adult mammalian brain, neurogenesis occurs in a few selected regions, such as the hippocampus, subventricular zone, and olfactory bulb (Moreno-Jiménez et al., 2019, 2021). The rodent hippocampus generates new neurons throughout life (Kempermann et al., 2018), and recent evidence in humans indicates that hippocampal neurogenesis is likely to persist throughout adulthood but declines with age (Babcock et al., 2021). Adult hippocampal neurogenesis is a striking form of neural plasticity that occurs in the brains of numerous mammalian species. It is regulated by several lifestyle factors, including exercise, diet, and social interactions (Valero et al., 2016; Augusto-Oliveira and Verkhratsky, 2021). Augusto-Oliveira and Verkhratsky (2021) reviewed how the adoption of a healthy lifestyle, including regular exercise, intellectual engagement, and friendly diet, impacts brain physiology from a molecular biological perspective. Lifestyle factors are associated with arresting or retarding neurodegenerative alterations, for example, exposure to an enriched environment results in well-characterized beneficial effects on the central nervous system, including boosting adult neurogenesis, synaptic plasticity, cellular physiology, and remodeling of the neuroglia.

## Brain Morphology and Age-Related Hearing Loss

There is growing evidence of a link between structural brain findings and hearing loss with aging (Lin et al., 2014; Qian et al., 2017; Rigters et al., 2017, 2018; Uchida et al., 2018; Armstrong et al., 2019, 2020a,b; Xu et al., 2019; Ha et al., 2020; Isler et al., 2021). Various studies have reported results using different assessments of brain structure and auditory function; the heterogeneity of study designs makes it difficult to discuss them in a unified manner (Jafari et al., 2021). Here, we focus on MRI-based studies that deal with populations with a reasonably large number of patients, since neuroimaging with MRI provides an accurate and reproducible assessment. In addition to the mainstream measurement of gray matter (GM) morphology and volume, there is also interest in the microstructure of the white matter (WM). Diffusion tensor imaging (DTI) is highly sensitive in detecting changes in WM microstructure, which provides an excellent marker for microstructural alterations before they can be identified using conventional MR methods (Jafari et al., 2021).

Even when limited to MRI-based studies, the main research targets are wide-ranging, such as the auditory cortex, including the primary auditory cortex, whole brain volume, GM, WM, and non-auditory cortex (Manno et al., 2021). Manno et al. (2021), conducted a comprehensive systematic review, meta-analysis, and meta-regression of the structural alterations of the brain due to hearing loss, and assessed the impact on the brain of pediatric, adult, and aged adult populations, to identify whether the etiology is congenital or acquired. Manno et al. (2021), stated that the impact of hearing loss on the brain was multifocal and not limited to the temporal lobe and that hearing loss was found to affect the GM and the underlying WM in nearly every region of the brain and affected all populations.

From a survey of aged adults, several reports with cross-sectional or longitudinal analyses have been published from large-scale epidemiological studies in various countries. Although this was a narrative review and an objective systematic method of article extraction was not used, the literatures were hand-selected to be as recent as and as large a study population as possible, using the keywords brain volume, brain morphology, MRI, WM, GM, auditory, aged, aging, age-related hearing impairment, presbycusis, hearing, hearing loss, and so on. Representative studies with more than 100 participants that addressed the association between hearing loss and brain volume using MRI are listed in Table 1.

**TABLE 1:** Description of studies with more than 100 subjects that addressed the association between hearing loss and brain volume using structural magnetic resonance imaging (MRI).

Studies		Research country/area	Design	Participants	Major findings
Uchida et al., 2018	NILS-LSA	Japan	Cross-sectional analysis	*n* = 2,082 Mean age 61.0 years Age range 40–89 years	More degraded peripheral hearing that was assessed by pure-tone audiometry was significantly correlated with smaller hippocampal volume after adjusting for potential confounding factors, and the association was consistent through the auditory frequency ranges. Hippocampal volume had a significant relationship with hearing level, with the standardized partial regression coefficients of −0.0758 for the speech range (*p* = 0.0029).

Lin et al., 2014	BLSA	United States	Longitudinal analysis (mean follow-up period: 6.4 years)	*n* = 126 Mean age not shown Age range 56–86 years	Compared to individuals with normal hearing, those with hearing impairments exhibited accelerated volume declines in the right temporal lobe (superior, middle, and inferior temporal gyri, and parahippocampus, *p* < 0.05).

Armstrong et al., 2019	BLSA	United States	Longitudinal analysis (mean duration between midlife hearing assessment and late-life MRI: 19.5 years)	*n* = 194 Mean age at hearing assessment, 54.5 years Age range not shown	Significant associations were found between poorer midlife hearing in the better ear and steeper late-life volumetric declines in the right temporal gray matter, right hippocampus, and left entorhinal cortex.

Armstrong et al., 2020b	BLSA	United States	Longitudinal analysis (a mean follow-up time: 1.7 years)	*n* = 356 Mean age 73.5 years Age range 55–99 years	Poorer peripheral hearing was associated with increases in mean diffusivity in the inferior fronto-occipital fasciculus and the body of the corpus callosum, but there were no associations of peripheral hearing with fractional anisotropy changes in these tracts. Poorer central auditory function was associated with longitudinal mean diffusivity increases and fractional anisotropy declines in the uncinate fasciculus.

Rigters et al., 2017	Rotterdam Study	The Netherlands	Cross-sectional analysis	*n* = 2,908 Mean age 64.9 years Age range 52–99 years	Hearing impairment was associated with a smaller total brain volume. Specifically, white matter volume was associated with hearing impairment, and this association was present in all the brain lobes. The associations were more pronounced in the lower frequencies of the pure-tone threshold.

Rigters et al., 2018	Rotterdam Study	The Netherlands	Cross-sectional analysis	*n* = 2,562 Mean age 69.3 years Age range not shown	Poorer white-matter microstructure in the right superior longitudinal fasciculus and the right uncinate fasciculus was significantly associated with worse hearing. These associations did not differ significantly between middle-aged (51–69 years) and older (70–100 years) participants.

Armstrong et al., 2020a	Rotterdam Study	The Netherlands	Cross-sectional analysis	*n* = 2,386 Mean age 64.8 years Age range 51.8–97.8 years	The association between the degrees of auditory speech processing performance (normal, insufficient, and poor) and brain volumes was examined cross-sectionally after pure-tone average adjustment, the degrees of auditory performance were not associated with brain volumes.

Rudner et al., 2019	UK Biobank	The United Kingdom	Cross-sectional analysis	*n* = 8,701 Mean age 62.3 years Age range not shown	Lower gray matter volume in both the auditory processing regions in the temporal cortex and the cognitive processing regions in the frontal cortex, as well as lower hippocampal volume, are associated with poorer ability to recognize speech in noise.

Xu et al., 2019	ADNI	North America	Longitudinal analysis	Subjects were evaluated for cortical thickness or volume measures of hippocampus Hearing loss group *n* = 131 mean age 77.5 years, age range not shown Hearing normal group *n* = 746 Mean age 73.0 years, age range not shown	Results of the longitudinal analyses showed that ARHL at baseline was associated with more rapid cortical thinning in the hippocampus. Hippocampus displayed significantly accelerated atrophy in individuals with ARHL (*p* < 0.01).
					

The Baltimore Longitudinal Study of Aging (BLSA) analyzed brain volume measurements performed with semi-automated region-of interest (ROI) algorithms of individuals with normal hearing versus those with hearing impairment (speech-frequency pure tone average > 25 dB) followed for a mean of 6.4 years after the baseline scan (*n* = 126, age 56–86 years) (Lin et al., 2014). The study concluded that hearing-impaired individuals had faster declines in the brain volume over time compared with that of their counterparts. Whole brain volumes declined by 8.4 versus 7.2 cm^3^/year, respectively, in those with hearing impairment versus normal hearing (*p* = 0.017). Individuals with hearing impairments exhibited accelerated volume declines in the whole brain and regions in the superior, middle, and inferior temporal gyri and parahippocampal gyrus of the right but not the left temporal lobe.

Armstrong et al. (2019), reported the results of a longitudinal analysis of the BLSA with a long period of follow-up (mean follow-up time, 19.5 years). A total of 194 community-dwelling older adults who had midlife measures of peripheral hearing at a mean age of 54.5 years and late-life volume change of up to 6 years between the first and most recent MRI assessments were studied. Poor midlife hearing in the better ear and steep late-life volumetric declines in the right temporal GM [β = −0.113; 95% confidence intervals (CIs), −0.182 to −0.044], right hippocampus (β = −0.008; 95% CI, −0.012 to −0.004), and left entorhinal cortex (β = −0.009; 95% CI, −0.015 to −0.003). Many associations were found between hearing impairment and great ventricular enlargement and annual volume loss in the total brain, lobar GM and WM regions, right middle and inferior temporal gyri, and left hippocampus.

From a longitudinal analysis of the BLSA, an association between hearing and changes in WM microstructure has also been published (Armstrong et al., 2020b). Three hundred and fifty-six cognitively normal adults (age range: 55–99, mean age: 73.5 ± 8.8 years) who had at least one hearing assessment and serial MRI session with DTI were evaluated with a mean follow-up time of 1.7 years. Poor peripheral hearing measured by pure-tone average in the better-hearing ear was associated with changes in mean diffusivity in the inferior fronto-occipital fasciculus and body of the corpus callosum. Poor central auditory function, measured by signal-to-noise ratio score from a speech-in-noise task, was associated with changes in the uncinate fasciculus. Armstrong et al. (2020b) interpreted that poor hearing was related to changes in the integrity of specific WM regions involved in auditory processing.

In the Rotterdam Study, a prospective cohort study ongoing since 1990 in the city of Rotterdam in the Netherlands comprising adults aged 45 years and older, the association between age-related hearing loss (ARHL) and morphological brain assessments has been investigated.

Rigters et al. (2017), examined the relationship between hearing impairments and brain volume using MRI in the Rotterdam Study, which included 2,908 participants (mean age: 64.9 years; 56% females). Global and regional brain tissue volumes (total brain volume, GM volume, WM volume, and lobe-specific volumes) were quantified. Rigters et al. (2017), quantified hearing impairments for the best hearing ear by taking the average threshold over all frequencies, namely 0.25, 0.50, 1, 2, 4, and 8 kHz. The results showed that hearing impairments were associated with a small total brain volume, which was driven by small WM volumes, which was consistent across the hearing frequencies but pronounced at low frequencies.

In the Rotterdam Study, the relationship between brain morphology and central auditory speech processing, as assessed with the Digits-in-Noise task, as well as peripheral auditory function, was also investigated (Rigters et al., 2018; Armstrong et al., 2020a). Rigters et al. (2018), quantified hearing acuity in 2,562 participants (mean age: 69.3 years) and reported that altered WM microstructure was associated with poor hearing on the pure-tone audiogram and digit-in-noise test, which reflected central auditory processing and cognitive skills. A poor WM microstructure was associated with poor hearing acuity, specifically in the right superior longitudinal fasciculus and uncinate fasciculus.

The speech recognition threshold and neuroimaging assessments (brain volumes and WM microstructure, measured with MRI and DTI, respectively) were analyzed by Armstrong et al. (2020a) cross-sectionally in 2,386 Rotterdam Study participants (age range: 51.8–97.8 years, mean age: 64.8 years). Brain volumes were assessed on a global and lobar level for specific dementia-related structures (the hippocampus, entorhinal cortex, and parahippocampal gyrus). A poor ability to understand speech in noise was associated with a large parietal lobe volume but not with DTI measures. When examining the association between the degree of auditory speech processing performance (normal, insufficient, and poor) and brain volumes cross-sectionally after pure-tone average adjustment, the degree of auditory performance was not associated with brain volume. Armstrong et al. (2020a) discussed why they did not replicate findings from a previous study of the UK Biobank (participants’ age range: 40–69 years, mean age: 62.3 years) (Rudner et al., 2019). Rudner et al. (2019), found that poor central auditory speech processing, as defined by the Digits-in-Noise summary score, was associated with low GM volumes in the data available from the UK Biobank Resource. While both studies used Digits-in-Noise to define central auditory speech processing among participants of similar age range, Rudner et al. (2019), did not adjust the models by continuous pure-tone average. Since the pure-tone average is a major factor that can confound the relationship between central auditory processing and brain structure, Armstrong et al. (2020a) discussed the necessity of including this factor in models that examine this relationship.

We published our results of analyzing the relationship between hearing ability assessed using pure-tone audiometry and the volume of brain regions, specifically focusing on the volumes of the hippocampus, Heschl’s gyrus, and total GM, using Freesurfer software and T1-weighted brain MRI in community dwellers in the National Institute for Longevity Sciences, Longitudinal Study of Aging (NILS-LSA) (Uchida et al., 2018). The data of 2,082 participants aged 40 years and older (age range: 40–89 years, mean age: 61.0 years) were extracted and analyzed cross-sectionally with adjustment for possible confounding factors. Individuals with hearing impairment showed significantly smaller hippocampal volumes for all auditory frequency ranges, compared with that of their counterparts without hearing impairment. A correlational analysis indicated a significant dose-response relationship between hearing acuity and hippocampal volume, consistent through the auditory frequency ranges. The volume of the left Heschl’s gyrus showed a significant relationship with hearing levels for some auditory frequencies. In the entorhinal cortex, right Heschl’s gyrus, and total GM, the volume did not correlate with hearing level at any frequency.

From the Alzheimer’s Disease Neuroimaging Initiative (ADNI) database, participants with ARHL were selected and analyzed cross-sectionally and longitudinally to explore how ARHL can influence cortical structure and several neurodegenerative biomarkers, such as the cerebrospinal fluid (CSF) β-amyloid (Aβ) and tau measurements, and brain Aβ load (Xu et al., 2019). Although hearing function was not quantified, data were extracted based on search terms, including “hear,” “auditory,” “ear,” “deaf,” “presbycusis,” and “hard of hearing,” on the medical history and physical examination records. The volume/thickness of the hippocampus and entorhinal cortex (*p* < 0.01 hippocampus; *p* < 0.05, entorhinal cortex) displayed significantly accelerated atrophy in individuals with ARHL, although the baseline volume/thickness of these two regions was high in individuals with ARHL. ARHL was associated with high CSF levels of total tau (*p* < 0.001) or ptau_181_ (*p* < 0.05) at the baseline and fast elevation rates of these two types of biomarkers (*p* < 0.05).

The regions that have been studied in relation to hearing loss using brain volume are quite diverse, and the results regarding their relationship are inconsistent. The causal relationship between hearing loss and increased risk of developing dementia is yet to be clarified, despite numerous epidemiological studies on the relationship between cognitive function and hearing loss. This may be another factor for a wide range of targets for researchers.

There are a number of possible mechanisms for the relationship between hearing loss and dementia (Lin and Albert, 2014; Fulton et al., 2015; Wayne and Johnsrude, 2015; Stahl, 2017; Chern and Golub, 2019; Uchida et al., 2019), and Griffiths et al. (2020), grouped them into four representative mechanisms that are not mutually exclusive. They appraised mechanisms based on a common pathology in the cochlea and brain, brain resources deterioration because of an impoverished acoustic environment, and the diminished availability of cognitive resources that are occupied in support of listening during difficult conditions, and proposed a novel mechanism that is based on a critical interaction between auditory cognitive processing in the medial temporal lobe (MTL) and dementia pathology. The role of MTL in auditory processing was introduced in detail by supportive results from many studies, including animal models, although MTL structures are not classically regarded as part of the auditory system. Animal studies have demonstrated neural outcomes of reduced auditory inputs, such as morphological changes throughout the auditory pathways, decreased cell density, impaired hippocampal neurogenesis, and a decrease in hippocampal synapses (Liu et al., 2016; Chang et al., 2019). Griffiths et al. (2020), favored the mechanism of interaction between neuronal activity and AD pathology in the MTL. This mechanism is supported by circumstantial evidence of the co-occurrence of altered neuronal activity due to hearing loss and AD pathology in the MTL. During difficult listening, a specific interaction with the molecular basis of AD can occur in the MTL. Early audiometric hearing loss was reported to be associated with the presence of brain β-amyloid, measured using positron emission tomography scans (Golub et al., 2021). In the analysis of acoustic patterns during speech-in-noise perception, an altered activity of auditory cognitive mechanisms has been reported, and studies support the involvement of the hippocampus in the analysis of degraded speech (Bishop and Miller, 2009; Blank et al., 2018). Human studies suggesting the use of computational mechanisms in the MTL for the active analysis of acoustic patterns were outlined by Griffiths et al. (2020), It was assumed that the interaction between increased activity and synaptic and/or molecular changes associated with AD occurs through the possible changes driven by hearing loss in MTL neural mechanisms.

## The Role of Hearing Management From the Perspective of the Relationship With the Brain

Griffiths et al. (2020), also discussed how the predicted effects of hearing intervention differ by mechanisms. If a common cause that affects the cochlea and/or the ascending pathway (causing hearing loss) and MTL (causing dementia), restoring hearing would not affect the development of dementia or lead to any improvement in cognition. In the other three mechanisms, the effect of hearing restoration on dementia risk reduction may be expected. In the aforementioned mechanism of interaction between brain activity related to auditory cognition and dementia pathology, early hearing restoration could reduce the risk by restoring normal activity to the hippocampus; otherwise, if the delay between initial hearing loss and remediation is too long, a chain of events may already have been set in motion to cause ongoing cortical degeneration after hearing restoration. The beneficial effect of hearing intervention may vary depending on the length of time between difficult listening and start of hearing aid use.

Johnson et al. (2021), summarized the structural and functional features of auditory brain organization that confer vulnerability to neurodegeneration, including the extensive, reciprocal interplay between the “peripheral” and “central” hearing dysfunction, and suggested that hearing impairment might plausibly constitute a proximity marker for incipient cognitive decline and dementia. As a countermeasure, Johnson et al. (2021), mentioned strategies involving novel auditory “cognitive stress tests” for detecting the early stages of neurodegeneration in population-based screening and recruitment of affected populations into dementia prevention trials. They pointed out that management approaches which focus solely on peripheral sound amplification are likely to have limited efficacy for improving hearing function in dementia, since neurodegenerative pathologies target the auditory brain and are therefore predicted to damage hearing function early and profoundly.

Hearing aids are an effective strategy for auditory rehabilitation and are the primary choice in individuals with ARHL. According the World Health Organization Guideline “Risk Reduction of Cognitive Decline and Dementia,” there is currently insufficient evidence to recommend the use of hearing aids to reduce the risk of cognitive decline/dementia, but the use of hearing aids is important for correcting hearing loss in older adults for other benefits [World Health Organization [WHO], 2019]. The neurophysiological mechanisms underlying hearing aid use remain unclear. Few studies have used radiological, physiological, or molecular pathological approaches to assess the effects of hearing aid use on cognition, but some attempts have been reported (Giroud et al., 2017; Pereira-Jorge et al., 2018; Rudner et al., 2019). Di Stadio et al. (2021), discussed the effects of hearing aids on the prevention and treatment of cognitive decline in the elderly by referring to previous studies. Pereira-Jorge et al. (2018), reported a valuable evidence that 1 year of hearing aid use is related to functional and anatomical brain changes extending to multimodal cortices.

There are no drug treatments that can cure AD or any other common type of dementia. Based on the recent evidence that neurogenesis in the human hippocampus likely persists throughout adulthood, and the fact that there are no significant adverse events associated with the use of hearing aids, hearing aid use in adults with ARHL is a promising option for maintaining cognitive function that deserves attention.

## Author Contributions

YU, YN, RO, and SS contributed to the search and assessment of the available literature. YU mainly wrote the manuscript. MS, TY, TK, KI, and AN interpreted the results of previous studies. All authors approved the final version of the manuscript before submission.

## Conflict of Interest

The authors declare that the research was conducted in the absence of any commercial or financial relationships that could be construed as a potential conflict of interest.

## Publisher’s Note

All claims expressed in this article are solely those of the authors and do not necessarily represent those of their affiliated organizations, or those of the publisher, the editors and the reviewers. Any product that may be evaluated in this article, or claim that may be made by its manufacturer, is not guaranteed or endorsed by the publisher.
